# Targeting the gut microbiota and its metabolites for type 2 diabetes mellitus

**DOI:** 10.3389/fendo.2023.1114424

**Published:** 2023-05-09

**Authors:** Jiaqiang Wu, Kangping Yang, Hancheng Fan, Meilin Wei, Qin Xiong

**Affiliations:** ^1^ The Second Clinical Medical College of Nanchang University, Second Affiliated Hospital of Nanchang University, Nanchang, China; ^2^ Department of Histology and Embryology, School of Basic Medicine, Nanchang University, Nanchang, China; ^3^ Department of Endocrinology and Metabolism, Second Affiliated Hospital of Nanchang University, Nanchang, China; ^4^ Department of Endocrinology and Metabolism, First Affiliated Hospital of Nanchang University, Nanchang, China; ^5^ Jiangxi Clinical Research Center for Endocrine and Metabolic Disease, Nanchang, China; ^6^ Jiangxi Branch of National Clinical Research Center for Metabolic Disease, Nanchang, China

**Keywords:** type 2 diabetes mellitus, gut microbiota, gut microbial metabolites, targeted therapy, probiotics

## Abstract

Type 2 diabetes mellitus (T2DM) is a metabolic disorder characterized by hyperglycemia and insulin resistance. The incidence of T2DM is increasing globally, and a growing body of evidence suggests that gut microbiota dysbiosis may contribute to the development of this disease. Gut microbiota-derived metabolites, including bile acids, lipopolysaccharide, trimethylamine-N-oxide, tryptophan and indole derivatives, and short-chain fatty acids, have been shown to be involved in the pathogenesis of T2DM, playing a key role in the host-microbe crosstalk. This review aims to summarize the molecular links between gut microbiota-derived metabolites and the pathogenesis of T2DM. Additionally, we review the potential therapy and treatments for T2DM using probiotics, prebiotics, fecal microbiota transplantation and other methods to modulate gut microbiota and its metabolites. Clinical trials investigating the role of gut microbiota and its metabolites have been critically discussed. This review highlights that targeting the gut microbiota and its metabolites could be a potential therapeutic strategy for the prevention and treatment of T2DM.

## 1 Introduction

Diabetes mellitus (DM) is considered one of the most serious public healthcare challenges in the world, with more than 536.6 million people aged 20-79 years (prevalence estimated at 10.5%) reported to have diabetes in 2021. This number is projected to rise to 783.2 million (prevalence estimated at 12.2%) by 2045 (1). Type 2 diabetes mellitus (T2DM), accounting for 90% of cases, is the most prevalent type and is characterized by hyperglycemia and insulin resistance (2, 3). The risk factors that contribute to the onset of T2DM are complex and have not been fully elucidated. Obesity, sedentary lifestyle, and genetic susceptibility are recognized as significant risk factors for T2DM progression (4). An increasing number of studies have shown a clear link between the dysregulated gut microbiota and the development of T2DM (5, 6). Understanding these interactions may lead to novel therapeutic implications for T2DM.

The gut microbiota is a complex and dynamic entity composed of trillions of microorganisms that live in close symbiosis with their host, consisting of hundreds of different species of bacteria, primarily distributed among nine phyla (7–9). It is dominated by the phylum *Firmicutes, Bacteroidetes, Proteobacteria, Actinobacteria, and Fusobacteria*, which account for 90% of the total human microbiota (10, 11). Gut microbiota is strongly influenced by geographic location, age, lifestyle, diet, and even the mode of birth (12–14). Furthermore, variations in gut microbiota can lead to changes in metabolites, such as bile acids (BAs), branched-chain amino acids (BCAA), short-chain fatty acids (SCFAs), lipopolysaccharides (LPS), trimethylamine (TMA), and propionic acid imidazole (PAI) (15). A study has demonstrated that an increase in trimethylamine-N-oxide (TMAO), a conversion product of TMA in the liver, predicts a high mortality risk in patients with T2DM (16). Although peripheral blood BAs levels do not predict the transition from impaired fasting glucose (IFG) to new-onset diabetes (NOD) (17), Tamara et al. reported a non-absorbable polymeric bile acids chelator (SAR442357) that ameliorated hyperglycemia in preclinical animal models of diabetes by reducing intestinal luminal bile acids levels and delaying the development of DM (18). These clinical reports suggest that gut microbiota and its metabolites may be significantly associated with T2DM progression (19, 20).

Current research generally concludes that gut microbial metabolites can influence the development of T2DM by modulating physiological processes such as β-cell dysfunction, chronic low-grade inflammation, oxidative stress, and dysmetabolism of lipids and glucose (21). For example, SCFAs can decrease the expression of pro-inflammatory cytokines by inhibiting NF-κB activation and IκBα degradation, improving glucose control, and mitigating the development of T2DM (22–24). Conversely, elevated TMAO levels impair glucose tolerance by blocking the hepatic insulin signaling pathway, causing systemic inflammation in adipose tissue, and accelerating the development of diabetes. Although these studies indicate that gut microbial metabolites play a role in the development of T2DM, a systematic summary of the molecular mechanisms involved is still lacking. This review aims to summarize the molecular links between microbiota-derived metabolites and the pathogenesis of T2DM, and discuss recent clinical trials and treatments for T2DM. A better understanding of the interactions between gut microbiota and T2DM could provide insights into T2DM prevention and therapy.

## 2 Diabetes and gut microbial metabolites

Gut microbial metabolites are compounds produced by gut microbiota during the digestion of food. These metabolites, including SCFAs, tryptophan metabolites, TMAO, LPS and BAs, have been shown to play a crucial role in the development of T2DM (25). Most of the metabolites can enter the systemic circulation and act as signaling molecules via various receptors, which further regulate multiple metabolic pathways.

### 2.1 Short-chain fatty acids

**Figure 1:**
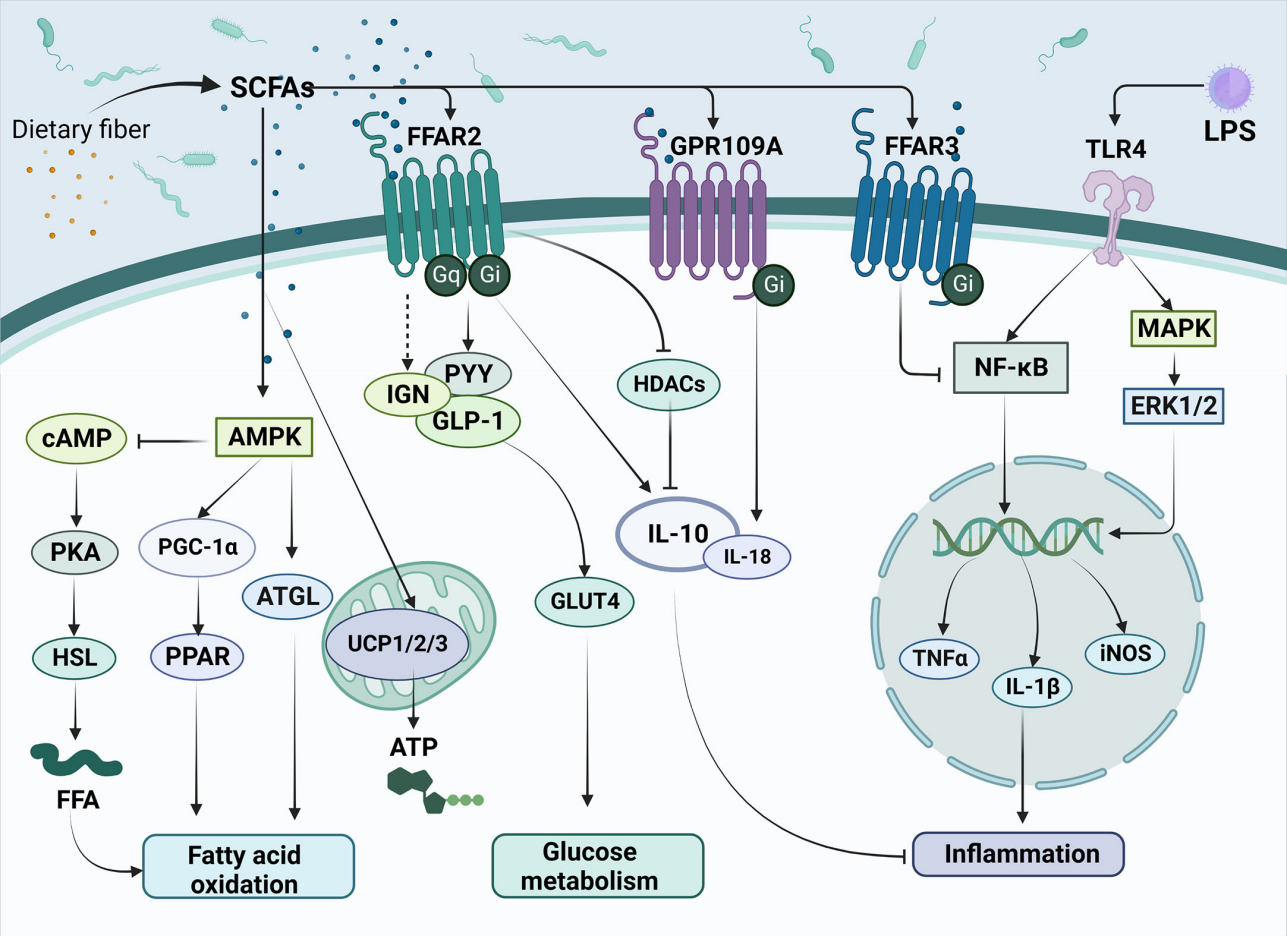
The main mechanisms of SCFAs regulating metabolism and inflammation in T2DM. SCFAs are produced by the conversion of dietary fiber by gut microbiota and subsequently enter cells directly or act on transmembrane receptors such as FFAR2, FFAR3 and GPR109A, which are involved in improving T2DM related pathways, such as fatty acid oxidation, glucose metabolism and inflammation response. Meanwhile, SCFAs can inhibit the release of inflammatory factors such as TNF-α and IL-1β triggered by LPS through the NK-κB pathway, thus alleviating the inflammatory response. SCFAs, short-chain fatty acids; FFAR2, Free Fatty Acid Receptor 2; FFAR3, Free Fatty Acid Receptor 3; GPR109A, G-protein-coupled receptor 109A; TLR4, Toll-like receptor 4; LPS, Lipopolysaccharide; AMPK, Adenosine 5’-monophosphate (AMP)-activated protein kinase; cAMP, Cyclic adenosine monophosphate; PKA, protein kinase A system; HSL, hormone-sensitive lipase; FFA, free fatty acid; PGC-1α, Peroxisome proliferator-activated receptor-γ coactivator-1α; PPAR, peroxisome proliferator activated receptor; ATGL, Adipose triglyceride lipase; UCP1/2/3, uncoupling protein1/2/3; ATP, Adenosine triphosphate; IGN, intestinal gluconeogenesis; PYY, peotide YY; GLP-1, glucagon-like peptide-1; GLUT4, glucose transporter 4; HDACs, Histone Deacetylases; IL-10, Interleukin-10; IL-18, Interleukin-18; NF-κB, nuclear factor kappa-B; MAPK, mitogen-activated protein kinase; ERK1/2, extracellular regulated protein kinases; TNFα, Tumor necrosis factor α; IL-1β, Interleukin-1β; iNOS, Inducible nitric oxide synthase.

SCFAs are major products of the anaerobic fermentation of resistant starch and fiber by the gut microbiota (**Figure 1**). Only a small fraction of SCFAs in the gastrointestinal tract is taken up from the diet. Butyric, acetic, and propionic acids constitute the most prevalent SCFAs in the body (26). SCFAs can be originally produced by thick-walled flora, including *Clostridium perfringens IV* and *XIV a*. These substances then enter the colonic epithelium via H-dependent or sodium-dependent monocarboxylate transport proteins to provide energy for their production (27). The remaining SCFAs that are then released from the intestine into circulation via the liver and portal system and contribute to the development of several diseases such as obesity, insulin resistance, T2DM, etc. (28).

As members of the fatty acid family, SCFAs can serve as substrates for lipid synthesis. It has been shown that SCFAs can activate AMPK, promote the induction of PGC-1α expression and activate peroxisome proliferator-activated receptor (PPAR), thereby regulating the fatty acid oxidation process (29, 30). Additionally, many studies have also pointed out that important lipid metabolic signals such as cAMP (31), adipose triglyceride lipase (ATGL, the main enzyme of lipolysis) (32), and uncoupling protein (UCP) (33) are also regulated by SCFAs. SCFAs have been found to play a role in hyperglycemic syndrome through G protein-coupled receptors (GPRCs) (34). The most crucial SCFAs receptors are the G protein-coupled receptors free fatty acid receptor 2 (FFAR2), free fatty acid receptor 2 (FFAR3), and G-protein-coupled receptor 109A (GPR109A). Extracellular signal-regulated kinase 1 or 2 (ERK1/2), intracellular calcium activation, cyclic adenosine monophosphate (cAMP), and G protein (Gq or Gi/o) are downstream signaling molecules that FFAR influences the absorption of nutrients (35, 36). FFAR2 (GPR43) is mainly expressed in white adipocytes, islet α and β cells, intestinal enteroendocrine cells, and immune cells (37, 38). Butyrate can inhibit histone deacetylase (HDAC) expression by activating FFAR2, thereby having an inhibitory effect on the inflammatory response (39). FFAR3 (GPR41) is expressed in white adipocytes, immune cells, pancreatic islet α and β cells, and intestinal enteroendocrine cells (40, 41). GPR109A is a G protein-coupled receptor for nicotinate and has poor sensitivity for butyrate (42, 43).

SCFAs have been extensively studied in the field of metabolic diseases. In a previous study, it was demonstrated that propionate upregulated peptide YY (PYY) and glucagon-like peptide-1 (GLP-1) expression in the colonic tissue, leading to weight loss and significantly reduced blood glucose levels (44). SCFAs activate FFAR2 on enteroendocrine L cells, thereby enhancing the release of GLP-1 and PYY (45). FFAR3 is expressed in vagal sensory neurons and cross-talks with cholecystokinin (CCK) to alter food intake (46). By regulating AMPK, GPR109A promotes Nrf2 nuclear import and induces autophagy, resulting in anti-inflammatory effect (47). It also regulates lipid metabolism and inhibits lipolysis in adipose tissue (48). A recent study also shows that SCFAs may contribute to the development of diabetes through DNA methylation (49).

### 2.2 LPS

Lipopolysaccharide (LPS) is an important feature on the cell wall of gram-negative bacteria and plays an important role in the pathogenesis of T2DM. LPS exhibits an interactive relationship with SCFAs (**Figure 1**) (50, 51). The amount of LPS can be used to predict the development of many inflammatory diseases associated with participation in natural immunity (52). It has been shown that ecological dysregulation due to high fat intake also upregulates LPS concentrations, resulting in the release of TNF, IL-1, and IL-6 and systemic inflammation (53). The development of endotoxemia will trigger the host’s immune response, entering a pro-inflammatory state, which may contribute to metabolic diseases, such as T2DM.

Toll-like receptor 4 (TLR-4) has been identified as an important receptor of LPS, which belongs to a family of transmembrane receptors. Upon TLR-4 activation, transcription of inflammatory cytokines such as TNF-α, IL-1, and IL-6 are enhanced via NF-κB and MAPK pathways. These inflammatory cytokines are significantly elevated in patients with T2DM, subsequently resulting in insulin resistance and pancreatic β-cell dysfunction (54).

### 2.3 Bile acids

**Figure 2:**
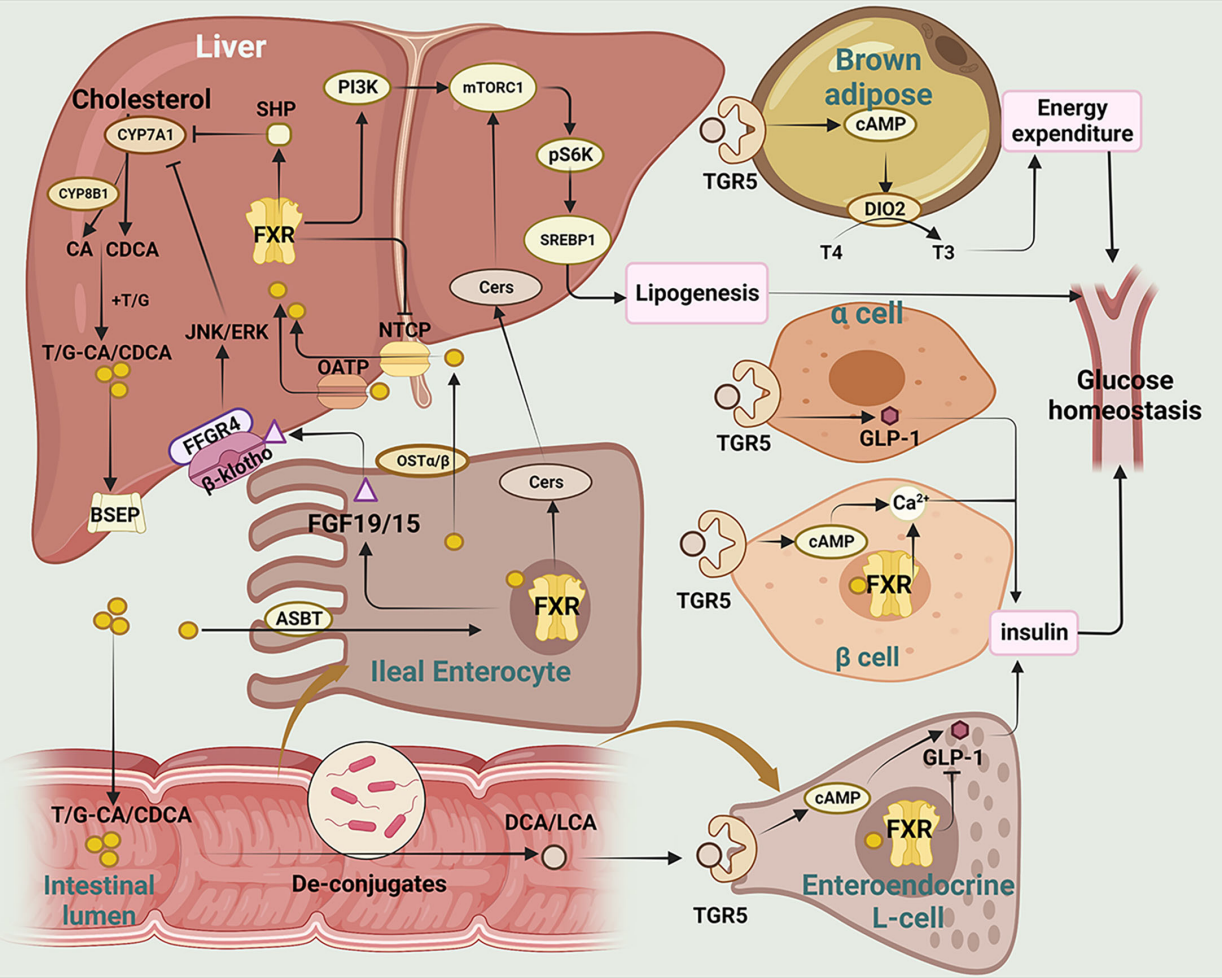
The main mechanisms of BAs regulating glucose homeostasis in T2DM. This figure illustrates the metabolism and transformation of bile acids in the liver, intestine, pancreas, and brown adipose tissue, and the mechanisms by which they regulate glucose homeostasis through the two major bile acid receptors, FXR and TGR5.CYP7A1, Cholesterol 7-alpha hydroxylase; CYP8B1, sterol 12α-hydroxylase; CA, cholic acid; CDCA, chenodeoxycholic acid; T/G, taurine/glycine; BSEP, bile salt export pump; SHP, small heterodimer; JNK/ERK, c-Jun N-terminal kinase/extracellular regulated protein kinases; FFGR4, FGF receptor 4; FGF19/15, fibroblast growth factor 19/15; ASBT, apical sodium-dependent bile acid transporter; PI3K, phosphatidylinositol-3-kinases; Akt, protein kinase B; mTOR, mammalian target of rapamycin; Cers,cermides;SREBP1, sterol-regulatory element binding proteins 1; FXR, farnesoid X receptor; NTCP, sodium taurocholate cotransporting polypeptide; OATP, Organic Anion Transporting Polypeptide; OSTα/β, organosolute transport proteins α and β; DCA, deoxycholic acid; LCA, lithic bile acids; TGR5, Takeda G protein-coupled receptor 5; cAMP, Cyclic adenosine monophosphate; DIO2, deiodinase type 2; T4, thyroxine; T3, triiodothyronine; GLP-1, glucagon-like peptide-1.

Bile acids (BAs), including chenodeoxycholic acids (CDCA) and cholic acids (CA), are synthesized in liver from cholesterol (55). There are two pathways of BAs synthesis: the classical pathway and the alternative pathway. CDCA is effectively catalyzed by mitochondrial sterol 27-hydroxylase (CYP27A1) for oxygenation of the carbon chain of corticosteroids, while the production of CA is determined by sterol 12-hydroxylase (CYP8B1) (56). The bile salts export pump (BSEP) then secretes bile salts that have been coupled with the amino-acid taurine or glycine into the digestive system, where they are converted into secondary BAs by gut microbiota. In the intestine, CA and CDCA can be converted into deoxycholic acids (DCA) and lithocholic acid (LCA) respectively by the action of bacterial bile salt hydrolase (BSH) and 7α-dehydroxylase enzyme (57). *Clostridium perfringens* is a bacterium that is capable of synthesizing the 7α-dehydroxylase enzyme (58). BSH is an enzyme produced by various strains of gut microbiota, including *Staphylococcus, Neococcus, Enterococcus, Bifidobacterium, Clostridium perfringens, and Parasiticum* (59). BAs are signaling molecules, which regulate insulin sensitivity and inflammation in T2DM via farnesoid X receptor (FXR) and Takeda G protein-coupled receptor 5 (TGR5) (**Figure 2**) (60–62). Meanwhile, BAs are also the ligands of vitamin D receptor (VDR) (63), progesterone X receptor (PXR) (64), membrane receptor sphingosine-1 phosphate receptor 2 (S1PR2) (65, 66) and play significant role in regulating inflammation and immune functions.

FXR is mainly expressed in the liver and intestine, and CDCA is the most potentially endogenous agonist of FXR (67). Activation of intestinal FXR induces the expression and secretion of fibroblast growth factor (FGF)15/19, which subsequently enters into liver via enterohepatic circulation (68). Serum FGF15/19 activates hepatic FGF receptor 4 (FGFR4)/-klotho complex, which in turn inhibits cholesterol 7-alpha hydroxylase (CYP7A1) transcription and reduces bile acid synthesis (69, 70). Additionally, it has been reported that clostridia-rich microbiota can promote BAs synthesis by suppressing intestinal FGF19 production (71). Activation of hepatic FXR promotes transcriptional activity of the small heterodimer (SHP), which in turn represses the expression of CYP7A1 expression and reduces bile acid synthesis. One of the downstream targets of FXR is insulin receptor substrate 1 (IRS1)-AKT-phosphatidylinositol 3 kinase (PI3K) pathway, which plays a crucial role in insulin signaling. While both intestinal and hepatic FXR signaling are involved in regulating bile acid homeostasis, they have distinct functions in lipids synthesis and absorption (72). Semi-synthetic bile acid, such as obeticholic acid (OCA), has been shown to be 30 times more effective in activating FXR than CDCA (73). OCA has been found to inhibit bile acid production, improve oxidative stress and liver fibrosis, and decrease hepatic cholesterol and triglyceride content (74, 75). In a study by Sunder et al. (NCT00501592), patients treated with 25 mg OCA showed that insulin sensitivity increased by 28.0% from baseline (76). These studies suggest that OCA may serve as a novel target in alleviating liver inflammation and insulin resistance.

TGR5 (also known as Gpbar-1) is a G protein-coupled receptor. TGR5 is widely expressed in various tissues, including the liver, adipose tissue and intestine, and plays important roles in regulating energy metabolism. TGR5 activation in enteroendocrine L cells can increase glucagon-like peptide-1 (GLP-1) secretion, leading to improved glucose homeostasis and insulin sensitivity (77). TGR5 activation in adipose tissue induces the expression of thyroid hormone deiodinase type 2 (DIO2), which converts inactive thyroxine (T4) into active thyroid hormone (T3) and enhances energy expenditure (78, 79).

Bile acid binding resins and bile acid chelator are used to regulate the BAs pathway (80). Bile acid binding resins work by binding to bile acids in the intestine, preventing their reabsorption and promoting their excretion in the feces. This leads to a reduction in the amount of bile acids in circulation, which in turn stimulates the liver to synthesize more bile acids from cholesterol. A clinical study of 40 Japanese patients with T2DM (NCT038934220) found that colestimide altered bile acid composition and increased the CA ratio, which enhanced energy metabolism, improved blood glucose levels, and alleviated diabetes via TGR5-cAMP-Dio2 pathway (81, 82). Berberine ursodeoxycholate (BUDCA) (83) has been shown to improve glycemic control and lower serum LDL-cholesterol level (NCT03656744) (84, 85). However, it should be noted that bile acid chelator may decrease the hydrophobicity of BAs and increase the risk of gallstone formation (86).

### 2.4 Tryptophan metabolites

**Figure 3:**
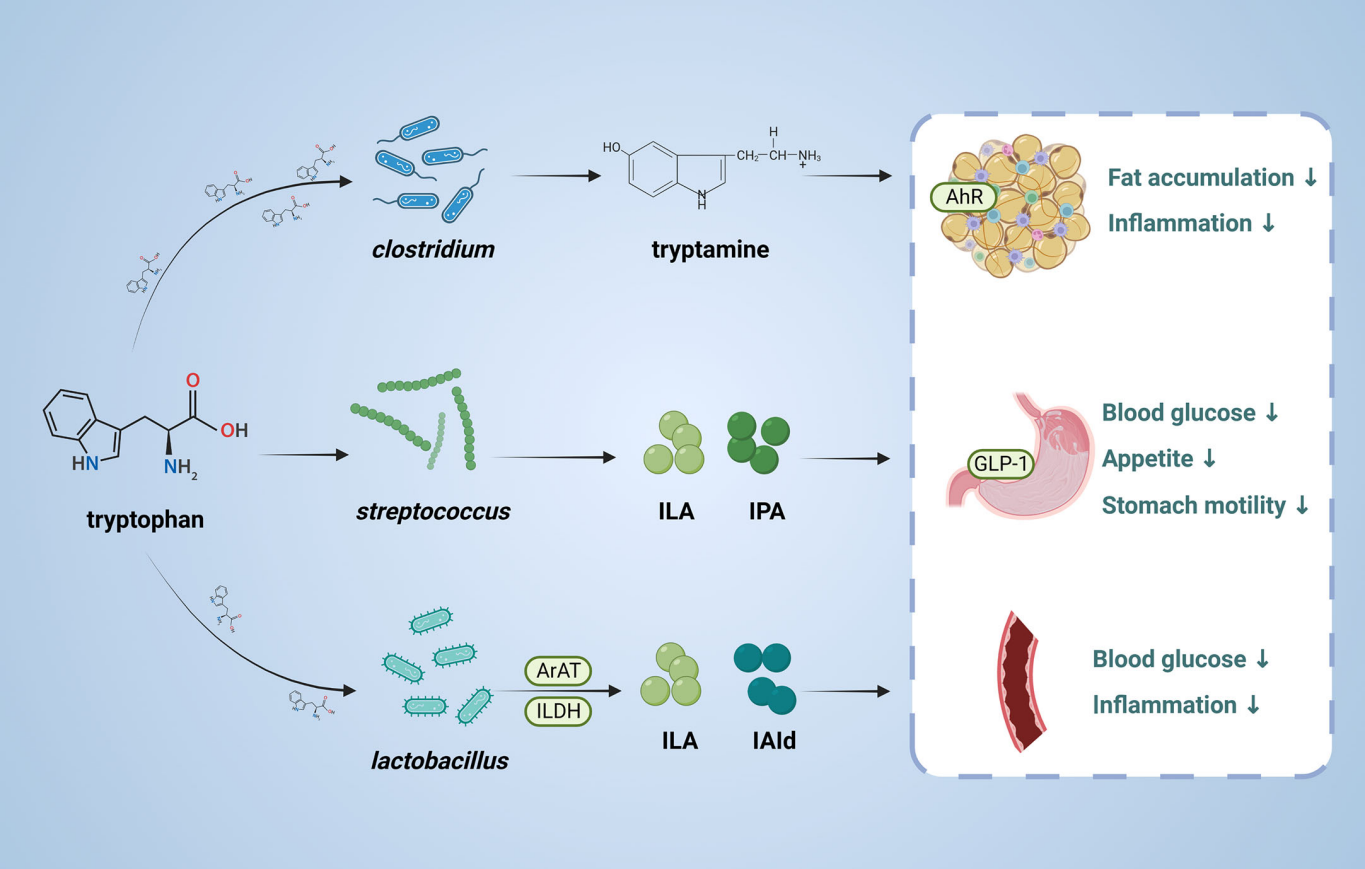
Role and mechanism of tryptophan metabolic pathway associated with T2DM. This figure depicts the conversion of tryptophan through the action of the gut microbiota, the products of which are involved in T2DM. ILA, indole-3-lactate; IPA, Indole 3-propionic acid;ILDH, indole-3-lactate dehydrogenase;ArAT Aromatic amino acid aminotransferase, IAld, indole-3-acetaldehyde; AhR, aryl hydrocarbon receptor; GLP-1, glucagon-like peptide-1.

Tryptophan is an essential amino acid and can be transformed by gut microbiota into molecules, such as indole and its derivatives, including indole-3- lactate (ILA), indole-3-propionic acid (IPA) and indole-3-acetaldehyde (IAld) (**Figure 3**). The tryptophan metabolites have been implicated in the pathogenesis of T2DM (87, 88). Indole stimulates GLP-1 secretion from intestinal L cells, resulting in insulin release and reduced blood glucose levels. IPA has been shown to have anti-inflammatory and antiseptic properties by acting on the aryl hydrocarbon receptor (AhR) (89–91). *In vivo*, administration of indole decreased hepatic steatosis and inflammation in rats fed high fat diet. And indole decreased lipid accumulation and stimulates inflammatory responses *in vitro* (92). Activation of AhR by tryptophan metabolites has been shown to have a variety of physiological effects, including regulation of immune responses, inflammation, and cell differentiation (93).

### 2.5 Trimethylamine N-oxide

Trimethylamine N-Oxide (TMAO) has been implicated in the pathogenesis of T2DM and related complications (94). Studies have shown that TMAO may contribute to the development of T2DM by promoting insulin resistance, impairing glucose tolerance, and inducing inflammation (95, 96). High level of TMAO may be associated with mild cognitive impairment, cardiovascular events in patients with T2DM (97–99).

TMAO is produced by gut bacteria from dietary nutrients such as egg and meat products (100). In intestine, gut microbiota breakdown choline, carnitine, or betaine into trimethylamine (TMA) and dimethylamine (DMA), which are absorbed into the bloodstream and transported to the liver (95).. In the liver, TMA and DMA are oxidized by the enzyme flavin-containing monooxygenase 3 (FMO3) to produce TMAO (101). Notably, a diet high in animal-based foods is associated with higher TMAO levels. The increase in circulating TMAO is thought to be possibly related to several factors, including: 1) dietary choline or carnitine content, 2) kidney function, 3) liver function, and 4) gut microbiota composition.

Many studies are focusing on regulating the TMA lytic enzymes to reduce TMAO levels. For example, 3,3-dimethyl-1-butanol (DMB), which is a structural analog of choline, can inhibit microbial TMA formation. It has been shown that DMB significantly reduces TMAO levels in mice fed a high choline or carnitine diet, thereby inhibiting diet-enhanced atherosclerosis (102). Metformin has also been found to decrease TMAO concentration in db/db mice (103). In addition, it has been demonstrated that berberine reduces TMAO levels by regulating TMA lytic enzymes via remodeling gut microbiota (104).

Antagonist against TMAO, another metabolite of the gut microbiota, has also been shown to improve glucose homeostasis and metabolism disorders (105). Taurisolo, a novel grape pomace polyphenolic extract, significantly decreased serum TMAO levels in healthy subjects (106). It implies that polyphenols can lower TMAO levels, thereby alleviating impaired glucose tolerance and improving adipose tissue inflammation in patients with T2DM (107).

TMAO has been implicated as a novel risk factor for cardiovascular events related to obesity and T2DM. Targeting gut microbiota and TMAO production may serve as potential therapeutic approaches for the treatment of T2DM.

## 3 Regulation of gut microbiota and its metabolites for T2DM therapy

**Figure 4:**
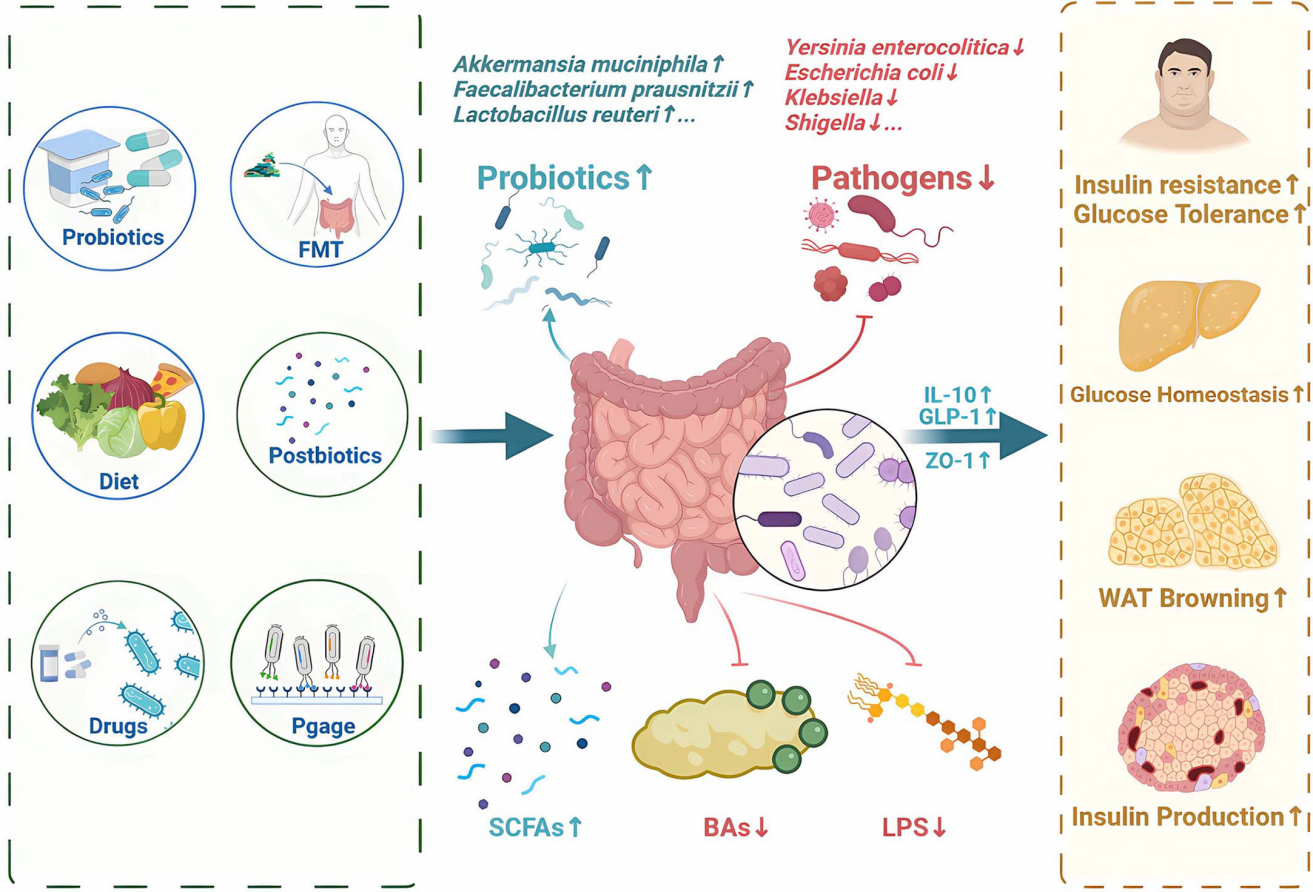
Potential therapy and treatments for T2DM by regulating gut microbiota and its metabolites. Recent approaches to regulate gut microbiota for T2DM therapy focuses on probiotics, prebiotics, synbiotics, fecal microbial transplantation, diet intervention, bacteriophages, microbiota-targeted drugs and postbiotics. SCFAs, short-chain fatty acids; FMT, Fecal Microbiota Transplantation; BAs,bile acids; LPS, Lipopolysaccharide; IL-10, Interleukin-10; GLP-1, glucagon-like peptide-1; ZO-1, zonula occludens-1.

As we gain a deeper understanding of the relationship between gut microbiota and T2DM, more and more therapies are emerging that aim to regulate the gut microbiota and its metabolites (**Supplementary Table 1**). Recent approach to regulate gut microbiota for T2DM therapy focuses on probiotics, prebiotics, synbiotics, fecal microbial transplantation, diet intervention, bacteriophages, microbiota-targeted drugs and postbiotics (**Figure 4**).

### 3.1 Probiotics, prebiotics, synbiotics

T2DM has been linked to dysbiosis of gut microbiota (108). Probiotics such as *Bifidobacterium, Lactobacillus*, prebiotics such as oligofructose and inulin, as well as synbiotics (a combination of the two) all play a significant role in the development of T2DM.

Probiotics are live microorganisms that provide beneficial effects to the host when adequately administered. Probiotics have been shown to improve glucose metabolism and insulin sensitivity in patients with T2DM. A combination of *Bifidobacterium lactis LMG P-28149* and *Lactobacillus rhamnosus LMG S-28148* increased PPARγ expression and enhanced insulin sensitivity in high-fat diet (HFD) induced obese mice (109). It has been shown that *Bifidobacterium longum* and *Lactobacillus* upregulated GLP-1 and IL-10 expression in patients with obesity or T2DM, and suppressed lipid accumulation in adipocytes (3, 110). In addition, *Lactobacillus fermentum MCC2760* increased the expression of glucose transporter 4 (GLUT4), GLP-1 and ZO-1, improving glucose tolerance in HFD mice (111).

Inulin is a type of prebiotic fiber that cannot be digested by the human body. It has been demonstrated that inulin is fermented by microbiota to produce SCFAs in the colon (112, 113). In a clinical trial (NCT02009670), consumption of inulin promotes SCFAs production and improves lipid oxidation, resulting in a significant improvement in glycemic control (114). Another study (NCT00750438) shows that inulin-propionate ester supplementation significantly increases colonic propionate levels, and prevents weight gain by promoting GLP-1 secretion (115).

Synbiotics, which combine probiotics and prebiotics, have the potential to provide more significant benefits than when used separately. For instance, when *Lactobacillus* paracasei *N1115* was combined with oligofructose, it was observed to down-regulate the expression of TLR4 and NF-κB, while up-regulating the p38 MAPK pathway (116). It is important to note that synbiotics currently lack FDA statements, and further clinical validation is required to determine the optimal ratio of probiotics and their safety and efficacy.

### 3.2 Fecal microbiota transplantation

Despite probiotics have been shown to have a potential role for T2DM, fecal microbiota transplantation (FMT) has advantage of entire gut microbiota transplantation. FMT has been recommended for the prevention of chronic *Clostridium difficile* infections since 2013, and it has also shown beneficial effects in ulcerative colitis and even metabolic diseases such as T2DM (117).

Studies have demonstrated that FMT treatment in mice reduces glucose levels, improves insulin sensitivity, and reduces islet cell apoptosis (117). Transplantation of normal human fecal flora into diabetic mice was reported to ameliorate glucose disorders by altering bacterial composition to produce more SCFAs and stimulating GLP-1 releasing via GPR43 receptor (118, 119). In contrast, mice transplanted with gut microbiota from patients with T2DM were found to disrupt blood glucose by regulating BAs metabolism (120). Study by Anne et al. reported that transplanting gut microbiota from lean donors to patients with T2DM could improve insulin sensitivity (121). Similarly, Su et al. showed that the predominant gut microbiota of T2DM patients shifted from *bacteroides* to *Prevotella* after FMT (122), with a significant increase in beneficial organisms (e.g., *bifidobacteria*) and a significant decrease in harmful organisms (e.g., *Bilobacteria*) in a 90-day open-label controlled trial (122). However, it should be noted that FMT may be ineffective or even cause side effects due to the complex composition of the gut microbiota. Elaine et al. reported that FMT had no clinically significant metabolic effects in a clinical study (NCT02530385) (123), possibly due to the small sample size of the trial. Adverse events such as diarrhea, constipation, abdominal pain, and infections have also been reported with FMT (124, 125).

Although FMT is a promising treatment for T2DM, more convincing evidence is needed to confirm the source of donors and frequency of FMT. The adverse effects of dangerous bacteria in the flora, the resilience of the gut microbiota, and the uncertain clinical result of microbiota modifications need more investigation (126, 127).

### 3.3 Diet interventions

A healthy diet helps patients with T2DM improve glycemic control. Research indicates that a weight loss about 15 kg induced by calorie restriction (CR) lead to remissions of T2DM in about 80% patients with obesity and T2DM (128, 129).

In the high-fat diet (HFD) group, an increase in LPS and TMAO and a decrease in SCFAs have been observed, which can affect the host metabolism and immunity. The significant elevation of *Escherichia coli*, *Klebsiella*, and *Shigella* in the HFD group and the decrease of *Lactobacillus* and *Lactobacillus* may provide an early warning for the development of T2DM. However, HFD can lead to an increase in the number of β-cells and induce a decrease of islet infiltration, protecting from the development of diabetes (130). This phenomenon may be related to impairment of immune checkpoints (ICPs) and reduced T-cell attack on pancreatic β-cells, which requires further investigation (131).

Interventions such as calorie restriction (CR), very low-calorie-ketogenic (VLCK), and fasting-mimicking diets (FMDs) have been utilized in metabolic diseases such as obesity and T2DM. CR was found to alter the microbiota and reprogram the metabolism, resulting in a different serum bile acid profile characterized by elevated ratio of non-12α-hydroxylated bile acids (132). The mechanism of CR induced glucose homeostasis may be related to GLP-1 secretion via TGR5/cAMP signaling pathway (133). Additionally, CR can reshape the gut microbiota composition and promote SCFAs production to exert anti-inflammatory effect. VLCK may induce elevating plasma concentrations of acetoacetic acid (ACA) and β-hydroxybutyric acid (β-OHB), and activation of white adipose tissue (WAT) lipolysis (134, 135). Ketogenic diets also alter the gut microbiota and reduce inflammatory Th17 cells (136). These studies indicate that more personalized diet interventions may be utilized for prevention and treatment of T2DM.

### 3.4 Bacteriophages

Gut microbiota contains not only bacteria but also a large number of viruses (dominated by bacteriophages) (137). Bacteriophages specifically infect bacteria in a host-specific manner and are associated with metabolic diseases. For example, altered viral taxonomic composition and reduced viral-bacterial correlation were observed in patients with obesity and T2DM (137, 138). In a previous study, the fecal virome from mice on a low-fat diet was transplanted into the intestine of mice on a high-fat diet. It was observed that the obese mice gained weight more slowly, and their glucose tolerance remained similar to that of mice on a low-fat diet (139). Bacteriophages therapy have been demonstrated to improve clinical healing of diabetic wounds and have less severe impact on the ecosystem than antibiotics (140).

A growing number of studies highlight the possibility that bacteriophages might modify their host genetics through the lysogenic pathway, leading to either an increase or decrease of metabolites levels. For instance, the abundance of *Klebsiella ph*age (*vB KpnP SU552A*) was found to be negatively correlated with tryptophan levels, indicating that targeting the tryptophan metabolic pathway by phages could regulate indole derivatives and potentially inhibit AhR to prevent insulin resistance (89–91, 141). However, further research is required to fully understand the role of bacteriophages in the treatment of T2DM, including larger clinical studies to confirm their efficacy.

### 3.5 Microbiota-targeted drugs

Microbiota-targeted drugs are a newly proposed class of drugs that aim to modulate the metabolites of gut microbiota. However, direct targeting of metabolites can have a significant effect on gastrointestinal function in clinical practice. Therefore, researchers are investigating how to target specific gut microbiota without affecting the gastrointestinal function.

Gut microbiota-derived metabolites play a central role in the host-microbe crosstalk (142, 143). Using a mini-gut model to screen drugs, THIP hydrochloride, methenamine, and mesna have been identified as promising new gut microbiota therapeutics (144). Amuc _1100, a specific outer membrane from *Akkermansia muciniphila*, has been shown to improve metabolism, insulin resistance and dyslipidemia (145, 146). THIP hydrochloride also has the effect of reducing the inflammatory response by decreasing the overgrowth of *Akkermansia muciniphila*, which can cause damage to the intestinal barrier. Urotropine significantly enhances the abundance of *Veillonellaceae*, which converts lactate into SCFAs (147). In addition, Mesna has been shown to decrease the number of *Verrucomicrobiaceae* and *Akkermansia muciniphila* while enhancing SCFA synthesis and decreasing endotoxin production. These changes may contribute to alleviating oxidative stress levels and chronic inflammation (148–150).

### 3.6 Postbiotics

Postbiotics are the byproducts of the metabolic processes of probiotic bacteria, including exopolysaccharides, γ-aminobutyric acid (GABA), and extracellular vesicles (EV) (151). For example, exopolysaccharide has been found to inhibit adipogenesis and pancreatic α-amylase by activating the AMPK signaling pathway (152, 153). It has been reported that GABA improves glucose intolerance, β-cell mass, and inflammatory response (154–156). EV from *Aeromonas aeruginosa* was found to improve intestinal barrier function and glucose tolerance in HFD-induced T2DM mice (157). Meanwhile, in this paper, we use a table (**Supplementary Table 2**) to summarize in as much detail as possible some information about completed or ongoing gut microbial metabolites clinical trials to show the latest progress of current gut microbial metabolites clinical studies.

## 4 Conclusion

In this review, we discuss the interaction between microbiota-derived metabolites and gut microbiota and their role in T2DM. Currently, there is growing interest in targeting the gut microbiota and its metabolites as a potential therapeutic approach for T2DM. Many approaches have been explored, including the use of probiotics, prebiotics, synbiotics, postbiotics, FMT, dietary interventions, bacteriophages, and microbiota-target drugs.

However, there are still several challenges that need to be addressed. One of the main challenges is the lack of a comprehensive understanding of the complex interactions between the gut microbiota, its metabolites, and the host. The gut microbiota is highly diverse and dynamic, and its composition can be influenced by various factors. Another challenge is the safety and efficacy of targeting the gut microbiota and its metabolites. Although there is growing evidence suggesting that targeting the gut microbiota and its metabolites can have beneficial effects on T2DM, there is also the potential for unintended consequences. In addition, better methods are needed to assess the gut microbiota and its metabolites. Current methods for assessing the gut microbiota and its metabolites, such as 16s rRNA sequencing, metagenomics and chromatography-mass spectrometry, have limitations in terms of resolution and accuracy. Finally, more high-quality clinical trials with larger sample size are needed to verify their safety and efficacy on T2DM. Taken together, a comprehensive understanding the interaction between microbiota-derived metabolites and T2DM will shed light into potential targets for T2DM therapy.

## Author contributions

Conceptualization and design: MW and QX. Writing-original draft preparation: JW and KY. Writing-review and editing: JW, KY and HF. Project administration: JW. Funding acquisition: MW and QX. Manuscript revised: MW and QX. All authors have read and agreed to the published version of the manuscript.
