# Supplementary material for: Targeting the gut microbiota and its metabolites for type 2 diabetes mellitus
Source: Front Endocrinol (Lausanne). 2023 May 9;14:1114424. doi: 10.3389/fendo.2023.1114424 (PMC10204722; doi:10.3389/fendo.2023.1114424)
Supplement: Supplementary file 1 [file Table_1.docx]

**Supplementary Table 1. Interventions and mechanisms for the therapy of T2DM through gut microbiota metabolites**

| **Type** | **Interventions** | **Doses** | **Main Effects and Mechanisms** | **Ref.** |
| --- | --- | --- | --- | --- |
| Probiotics | ProMetS probiotics powder | 4 g/day | Short B. breve, microbial lipid import and mobilization↑ | (1) |
|  | Lactobacillus acidophilus La-5 and lactis BB-12 | 10^9^ CFU/d, each | TNF-α↓, acetic acid↑ | (2) |
|  | WB-010(Akkermansia muciniphila, etc.) | 3 capsules administered twice daily | Insulin secretion ↑, improve blood glucose | (3) |
|  | Bifidobacterium longum PI10; Ligilactobacillus salivarius PI2 | 5 × 10^8^ CFU | GLP-1 and IL-10 ↑ | (4) |
| FMT | 4 Fecal donor samples | 1 g per capsule | The number of probiotics↑, dyslipidemia↓ | (5) |
|  | Rigorous anonymous screening of stool donor samples | unknown | D-arginine, D-ornithine metabolism↑and secondary BAs↑ | (6) |
| Diet | LCD and high-protein | 55% carbohydrates, 25% fat and 20% protein | Alteration of 10 microbial genera associated with glycemic variability | (7) |
|  | LCD | 130 g/day | Glucose and HbA1c↓, insulin resistance↓ | (8) |
|  | VLCDs of <800 kcal/d | 50% carbohydrate, 20% protein and 30% fat | Restore insulin sensitivity and insulin resistance↓ | (9) |
| Microbiota-targeted drug | THIP hydrochloride | Unknown | Interacts with TLR2 and the absorption of LPS↓ | (10) |
|  | Urotropine | 10^9^ CFU | lactic acid ↓and SCFAs ↑ | (11) |
|  | Mesna | 100 mg/kg per dose | SCFAs↑, LPS and blood glucose↓ | (12) |
| Postbiotics | Exopolysaccharide | 10^−8^Mol exopolysaccharide | GLUT-4, Akt-2 and AMPK ↑ | (13) |
|  | GABAs | 2.6 mg/kg body weight | Blood glucose and inflammatory responses↓ | (14) |
|  | EV | 10 μg per mice | Improve intestinal barrier function and glucose tolerance | (15) |
| Bacteriophages | vB KpnP SU552A | unknown | AhR and prevent insulin resistance↓ | (16) |

**Abbreviation:** CFU, colony-forming units; TNFα, Tumor necrosis factor α; GLP-1, glucagon-like peptide-1; IL-1β, Interleukin-1β; BAs, bile acids; VLCDs: very low calorie diets; LCD, low-carbohydrate diet; TLR2, Toll-like receptor 2; VLCDs: very low calorie diets; LPS, Lipopolysaccharide; SCFAs, short-chain fatty acids; Akt, protein kinase B; mTOR, mammalian target of rapamycin; GLUT-4, glucose transporter 4; AMPK, Adenosine 5‘-monophosphate (AMP)-activated protein kinase; EV, extracellular vesicle; AhR, Aryl hydrocarbon receptor.

**Reference**

1. Wang S, Ren H, Zhong H, Zhao X, Li C, Ma J, et al. Combined berberine and probiotic treatment as an effective regimen for improving postprandial hyperlipidemia in type 2 diabetes patients: a double blinded placebo controlled randomized study. Gut Microbes. 2022;14(1):2003176.

2. Tonucci LB, Olbrich Dos Santos KM, Licursi de Oliveira L, Rocha Ribeiro SM, Duarte Martino HS. Clinical application of probiotics in type 2 diabetes mellitus: A randomized, double-blind, placebo-controlled study. Clin Nutr. 2017;36(1):85-92.

3. Perraudeau F, McMurdie P, Bullard J, Cheng A, Cutcliffe C, Deo A, et al. Improvements to postprandial glucose control in subjects with type 2 diabetes: a multicenter, double blind, randomized placebo-controlled trial of a novel probiotic formulation. BMJ Open Diabetes Res Care. 2020;8(1).

4. Alard J, Cudennec B, Boutillier D, Peucelle V, Descat A, Decoin R, et al. Multiple Selection Criteria for Probiotic Strains with High Potential for Obesity Management. Nutrients. 2021;13(3).

5. Su L, Hong Z, Zhou T, Jian Y, Xu M, Zhang X, et al. Health improvements of type 2 diabetic patients through diet and diet plus fecal microbiota transplantation. Sci Rep. 2022;12(1):1152.

6. Fujimoto K, Kimura Y, Allegretti JR, Yamamoto M, Zhang YZ, Katayama K, et al. Functional Restoration of Bacteriomes and Viromes by Fecal Microbiota Transplantation. Gastroenterology. 2021;160(6):2089-102.e12.

7. Tettamanzi F, Bagnardi V, Louca P, Nogal A, Monti GS, Mambrini SP, et al. A High Protein Diet Is More Effective in Improving Insulin Resistance and Glycemic Variability Compared to a Mediterranean Diet-A Cross-Over Controlled Inpatient Dietary Study. Nutrients. 2021;13(12).

8. Sato J, Kanazawa A, Makita S, Hatae C, Komiya K, Shimizu T, et al. A randomized controlled trial of 130 g/day low-carbohydrate diet in type 2 diabetes with poor glycemic control. Clin Nutr. 2017;36(4):992-1000.

9. Meehan CA, Cochran E, Mattingly M, Gorden P, Brown RJ. Mild Caloric Restriction Decreases Insulin Requirements in Patients With Type 2 Diabetes and Severe Insulin Resistance. Medicine (Baltimore). 2015;94(30):e1160.

10. Yan J, Sheng L, Li H. Akkermansia muciniphila: is it the Holy Grail for ameliorating metabolic diseases? Gut Microbes. 2021;13(1):1984104.

11. Scheiman J, Luber JM, Chavkin TA, MacDonald T, Tung A, Pham LD, et al. Meta-omics analysis of elite athletes identifies a performance-enhancing microbe that functions via lactate metabolism. Nat Med. 2019;25(7):1104-9.

12. Hagar HH, Almubrik SA, Attia NM, Aljasser SN. Mesna Alleviates Cerulein-Induced Acute Pancreatitis by Inhibiting the Inflammatory Response and Oxidative Stress in Experimental Rats. Dig Dis Sci. 2020;65(12):3583-91.

13. Huang Z, Lin F, Zhu X, Zhang C, Jiang M, Lu Z. An exopolysaccharide from Lactobacillus plantarum H31 in pickled cabbage inhibits pancreas α-amylase and regulating metabolic markers in HepG2 cells by AMPK/PI3K/Akt pathway. Int J Biol Macromol. 2020;143:775-84.

14. Marques TM, Patterson E, Wall R, O'Sullivan O, Fitzgerald GF, Cotter PD, et al. Influence of GABA and GABA-producing Lactobacillus brevis DPC 6108 on the development of diabetes in a streptozotocin rat model. Benef Microbes. 2016;7(3):409-20.

15. Chelakkot C, Choi Y, Kim DK, Park HT, Ghim J, Kwon Y, et al. Akkermansia muciniphila-derived extracellular vesicles influence gut permeability through the regulation of tight junctions. Exp Mol Med. 2018;50(2):e450.

16. Yang K, Niu J, Zuo T, Sun Y, Xu Z, Tang W, et al. Alterations in the Gut Virome in Obesity and Type 2 Diabetes Mellitus. Gastroenterology. 2021;161(4):1257-69.e13.
