# Supplementary material for: Targeting the gut microbiota and its metabolites for type 2 diabetes mellitus
Source: Front Endocrinol (Lausanne). 2023 May 9;14:1114424. doi: 10.3389/fendo.2023.1114424 (PMC10204722; doi:10.3389/fendo.2023.1114424)
Supplement: Supplementary file 2 [file Table_2.docx]

| **Category** | **NCT Number** | **Trial Description** | **Interventions** | **Country** | **Age** | **Phases** | **Enrollment** |
| --- | --- | --- | --- | --- | --- | --- | --- |
| **SCFAs** | [NCT02009670](https://clinicaltrials.gov/ct2/show/NCT02009670?term=NCT02009670&draw=2&rank=1) | Inulin,SCFA Production and Metabolic Response | 13C inulin | Netherlands | 20-50 | NA | 15 |
|  | [NCT01983046](https://clinicaltrials.gov/ct2/show/NCT01983046?term=NCT01983046&draw=2&rank=1) | Rectal SCFAs Combinations and Substrate and Energy Metabolism | Acetate, butyrate and propionate | Netherlands | 20-50 | NA | 12 |
|  | [NCT04209075](https://clinicaltrials.gov/ct2/show/NCT04209075?term=NCT04209075&draw=2&rank=1) | Prebiotics and Metformin Improve Gut and Hormones in T2DM in Youth (MIGHTY-fiber) | Biomebliss | US | 10-25 | Phase2 | 6 |
|  | [NCT05378295](https://clinicaltrials.gov/ct2/show/NCT05378295?term=NCT05378295&draw=2&rank=1) | Personalized Fiber and Insulin Sensitivity | Fermentable oligosaccharide | Netherlands | 30-70 | NA | 44 |
| **BAs** | [NCT00476710](https://clinicaltrials.gov/ct2/show/NCT00476710?term=NCT00476710&draw=2&rank=1) | Effects of Colesevelam HCl On BAs Kinetics | Colesevelam HCl | US | 40-60 | NA | 36 |
|  | [NCT02061124](https://clinicaltrials.gov/ct2/show/NCT02061124?term=NCT02061124&draw=2&rank=1) | Effect of BAs Sequestration on Postprandial GLP-1 Secretion, Glucose Homeostasis and gut microbiota | Sevelamer | Denmark | 35-80 | NA | 50 |
|  | [NCT01337440](https://clinicaltrials.gov/ct2/show/NCT01337440?term=NCT01337440&draw=2&rank=1) | Efficacy and Safety of UDCA Added to the DPP-4 Inhibitor in People With T2DM and Chronic Liver Diseases | UDCA/ Sitagliptin  Ursodeoxych-olic Acid | Japan | Child | Phase4 | 20 |
|  | [NCT01258075](https://clinicaltrials.gov/ct2/show/NCT01258075?term=NCT01258075&draw=2&rank=1) | Colesevelam for Children With T2DM (WEL Kid DM) | colesevelam | US | 10-17 | Phase4 | 236 |
|  | [NCT03462940](https://clinicaltrials.gov/ct2/show/NCT03462940?term=NCT03462940&draw=2&rank=1) | Effects of TUDCA on Endothelial Function in T2DM | Tauroursodeo-xycholic acid | US | 21-75 | NA | 2 |
| **LPS** | [NCT02186080](https://clinicaltrials.gov/ct2/show/NCT02186080?term=NCT02186080&draw=2&rank=1) | Effect of Gemigliptin on Metabolic Endotoxemia and Lipemia | Gemigliptin | Korea | 20-75 | NA | 10 |
| **TAMO** | [NCT03315988](https://clinicaltrials.gov/ct2/show/NCT03315988?term=NCT03315988&draw=2&rank=1) | The Effects of an 8-week Vegan Diet on TMAO Levels and Post-challenge Glucose in Individuals With Dysglycaemia | Vegan diet | UK | 18-75 | NA | 28 |
|  | [NCT03130894](https://clinicaltrials.gov/ct2/show/NCT03130894?term=NCT03130894&draw=2&rank=1) | Association Between TMAO and Diabetes | TMAO | China | 30-80 | NA | 2694 |
| **FMT** | [NCT05253768](https://clinicaltrials.gov/ct2/show/NCT05253768?term=NCT05253768&draw=2&rank=1) | Safety and Efficacy of Human FMT for Overweight and Obese Type 2 Diabetes Mellitus | FMT | China | 18 -65 | Phase2 | 40 |
|  | [NCT05076656](https://clinicaltrials.gov/ct2/show/NCT05076656?term=NCT05076656&draw=2&rank=1) | Epigenetic and Microbiota Modifications | FMT | Spain | 30-60 | Phase1 | 22 |
|  | [NCT03127696](https://clinicaltrials.gov/ct2/show/NCT03127696?term=NCT03127696&draw=2&rank=1) | Randomized Placebo-controlled Study of FMT to Impact Weight and Glycemic Control in Obese Subjects With T2DM | FMT | China | 18-70 | NA | 61 |
|  | [NCT03578991](https://clinicaltrials.gov/ct2/show/NCT03578991?term=NCT03578991&draw=2&rank=1) | T2DM as an Accelerator of Cognitive Impairment and Alzheimer's Disease | FMT | Spain | 65-85 | NA | 54 |
|  | [NCT04361097](https://clinicaltrials.gov/ct2/show/NCT04361097?term=NCT04361097&draw=2&rank=1) | FMT as a Therapeutic Strategy in the Progression of Chronic Kidney Disease | FMT | Mexico | ＞18 | NA | 28 |
| **Probiotic** | [NCT05418179](https://clinicaltrials.gov/ct2/show/NCT05418179?term=NCT05418179&draw=2&rank=1) | Effect of Probiotic Supplementation on Fecal Microbiota, Nutritional Status, Metabolic and Inflammatory Parameters | Probiotic | Brazil | 35-75 | NA | 80 |
|  | [NCT01765517](https://clinicaltrials.gov/ct2/show/NCT01765517?term=NCT01765517&draw=2&rank=1) | Study to Explore the Effects of Probiotics on Endotoxin Levels in T2DM Patients | Probiotic | Saudi Arabia | 20-75 | NA | 83 |
|  | [NCT02144948](https://clinicaltrials.gov/ct2/show/NCT02144948?term=NCT02144948&draw=2&rank=1) | Investigation of the Effect of E.-Coli-Nissle as Supporting Therapy to Standard Care of T2DM | e.-coli-nissle | Germany | 45-80 | Phase3 | 10 |
|  | [NCT05066152](https://clinicaltrials.gov/ct2/show/NCT05066152?term=NCT05066152&draw=2&rank=1) | The Effect of Single Probiotic on Metabolic Control in T2DM | ATCC 53103 | Turkey | 30-60 | NA | 34 |
|  | [NCT02861261](https://clinicaltrials.gov/ct2/show/NCT02861261?term=NCT02861261&draw=2&rank=1) | A Study on the Efficacy and gut microbiota of Berberine and Probiotics in Patients With Newly Diagnosed T2DM | ProMetS probiotics powder | China | 20-69 | Phase3 | 400 |
| **CR** | [NCT01930136](https://clinicaltrials.gov/ct2/show/NCT01930136?term=NCT01930136&draw=2&rank=1) | Effects of Caloric Restriction in Obesity and T2DM | CR (25%) | Italy | ＞40 | NA | 103 |
|  | [NCT01329822](https://clinicaltrials.gov/ct2/show/NCT01329822?term=NCT01329822&draw=2&rank=1) | Effects of Caloric Restriction on Fetuin-A and Cardiovascular Risk Factors | CR | Korea | 35-70 | NA | 76 |

**Supplementary Table 2. Clinical trials investigating the role of gut microbiota and its metabolites for T2DM**

**Abbreviation:** SCFAs, Short-chain fatty acids; T2DM, Type 2 diabetes mellitus; BAs, Bile acids; UDCA, Ursodeoxycholic acid; TUDCA, Tauroursodeo-xycholic acid; LPS, Lipopolysaccharide; TAMO, Trimethylamine-N-oxide; FMT, Fecal Microbiota Transplantation; CR, Caloric Restriction.
